# Reproductive outcomes following assisted reproductive technology after previous caesarean section: data from the Swiss National Registry

**DOI:** 10.3389/fendo.2026.1639111

**Published:** 2026-08-27

**Authors:** Angela Vidal, Jérémy Levy, Alexandra Kohl Schwartz, Michael von Wolff

**Affiliations:** 1 Division of Gynecological Endocrinology and Reproductive Medicine, Women’s University Hospital, Inselspital Bern, University of Bern, Bern, Switzerland; 2 Swiss Society for Reproductive Medicine, Aarau, Switzerland; 3 Department of Reproductive Endocrinology, Canton Hospital Lucerne, Lucerne, Switzerland

**Keywords:** ART, caesarean section, embryo transfer, pregnancy outcome, vaginal delivery

## Abstract

**Introduction:**

Caesarean section rates have risen worldwide, exceeding the rate of 10%–15% recommended by the WHO. This increase is driven by factors such as advanced maternal age, broader indications for caesarean section (CS), and increased use of assisted reproductive treatment (ART). Previous research, including meta-analyses, suggests that prior CS delivery (CSD) negatively affects the reproductive outcomes in ART, such as lower live birth rates (LBR) and potential subfertility, although the quality of the evidence is variable. Obstetric complications associated with CSD, for instance, uterine rupture and placental abnormalities, highlight the need for further investigation. This study therefore evaluates the impact of CSD on ART outcomes using a comprehensive national registry.

**Materials and methods:**

This study includes a cohort of 3,266 women who underwent caesarean section delivery (*n* = 1,363) or vaginal delivery (VD) (*n* = 1,903) followed by ART therapy with fresh or frozen embryo transfer (ET) from 2014 to 2022. The outcomes of the first ET and of all ETs after CSD or VD were compared using univariate analyses. Mixed logit models were applied for the outcome of the first ET. For the cumulative outcome of all ETs, mixed Cox models were used. The primary outcome was clinical pregnancy rate (CPR), and the secondary outcomes were LBR. Pregnancy outcome parameters included early pregnancy complications such as vaginal bleeding and miscarriages.

**Results:**

The characteristics of the patients in both study groups were similar. Following the first embryo transfer, the clinical pregnancy rates were lower after CSD in univariate analysis (38.7% vs. 43.4%; *p* = 0.008) but not statistically significant after multivariable adjustment significant (odds ratio (OR) = 0.87, 95% CI: 0.76–1.01). In contrast, cumulative analyses across all embryo transfers demonstrated significantly lower cumulative clinical pregnancy and live birth rates after CSD (hazard ratio (HR) = 0.89, 95% CI: 0.82–0.97, *p* = 0.009 and HR 0.89, 95% CI: 0.81–0.97, *p* = 0.010, respectively). Miscarriage rates and pregnancy complications were not increased by CSD.

**Conclusions:**

A history of caesarean section was associated with lower clinical pregnancy rates across successive embryo transfers following ART. No significant association was observed after the first embryo transfer following multivariable adjustment, and miscarriage rates were not affected by the mode of the previous delivery. Further prospective studies, including imaging assessment of caesarean scar defects and isthmocele, are required to determine whether the observed association is related to caesarean section itself or to secondary scar pathology.

## Introduction

Caesarean section (CSD) rates have increased worldwide (1, 2). Between 2000 and 2015, the global CSD rate nearly doubled from 12% to 21%, with rates of 32.8% in the United States, 32.4% in Australia, and 36.2% in China (3). In Switzerland, the prevalence of CSD was only 22% in 1998, but it has increased since then and reached a prevalence rate of 32% in 2021 (4). The global CSD rate is thus more than twice as high as the WHO recommendation of 10% to 15% (2, 5, 6).

The observed increased global CSD rate has been attributed to a number of factors, including increasing maternal age, an expansion of the indications for CSD, and a significant decrease in vaginal births after a previous CSD (2) as well as changes in women’s individual preferences and increased use of assisted reproductive technologies (ART) (2, 7, 8).

A potential association between mode of delivery and subfertility has important social, clinical, and public health implications (9, 10), particularly given that the global prevalence of infertility is estimated to range from 12.6% to 17.5% (11). The short- and long-term effects of caesarean section are not yet broadly well understood; however, one potential long-term consequence of the procedure is the development of an isthmocele, which is a defect at the site of the uterine scar (12–14). A correlation has been established between this defect and secondary infertility, attributable to factors such as menstrual blood retention, chronic inflammation, and diminished endometrial receptivity (6, 15–17). In the context of obstetric complications, there is a necessity to consider obstetric complications in future pregnancies, including caesarean scar pregnancy, placental abnormalities, uterine rupture, and postpartum hemorrhage (18). Notably, the risk of complications in subsequent pregnancies is associated with the mode of delivery.

The association between previous caesarean section delivery (CSD) and ART outcome had been investigated around 10 years ago. In 2013, a systematic review and meta-analysis found that many women with previous CSD had lower subsequent pregnancy and live birth rates (LBR) compared to those with previous vaginal delivery (VD) (5).

Riemma et al. (19) addressed this topic more recently in a systematic review and meta-analysis and showed in ART a reduction of LBR (RR 0.88, 95% CI: 0.79–0.99) but not of clinical pregnancy rate (CPR) after CSD compared to VD. They concluded that previous CSD seem to affect reproductive outcomes in ART. However, this conclusion was mainly based on large historical cohort studies, with low or very low grade of evidence.

As CSD rates rise worldwide and are nearly twice as high after ART compared to non-ART pregnancies (OR 1.9, 95% CI: 1.76–2.06) (20), their impact on reproductive outcomes has major public health implications given the short- and long-term consequences.

Most available studies are based on relatively small cohorts, historical ART populations, or selected patient groups, and several lacked adjustment for important confounders or comprehensive assessment of cumulative ART outcomes. Furthermore, evidence regarding obstetric outcomes after subsequent ART pregnancies remains scarce. These limitations justify a contemporary nationwide analysis using standardized registry data.

Therefore, the present study was designed not merely to reassess the association between previous caesarean delivery and ART outcomes but also to address several important limitations of the existing literature. Using a contemporary nationwide Swiss ART registry, including all IVF/ICSI treatments performed between 2014 and 2022, we evaluated both first-cycle and cumulative reproductive outcomes following previous caesarean versus vaginal delivery.

## Methods

**Figure 1:**
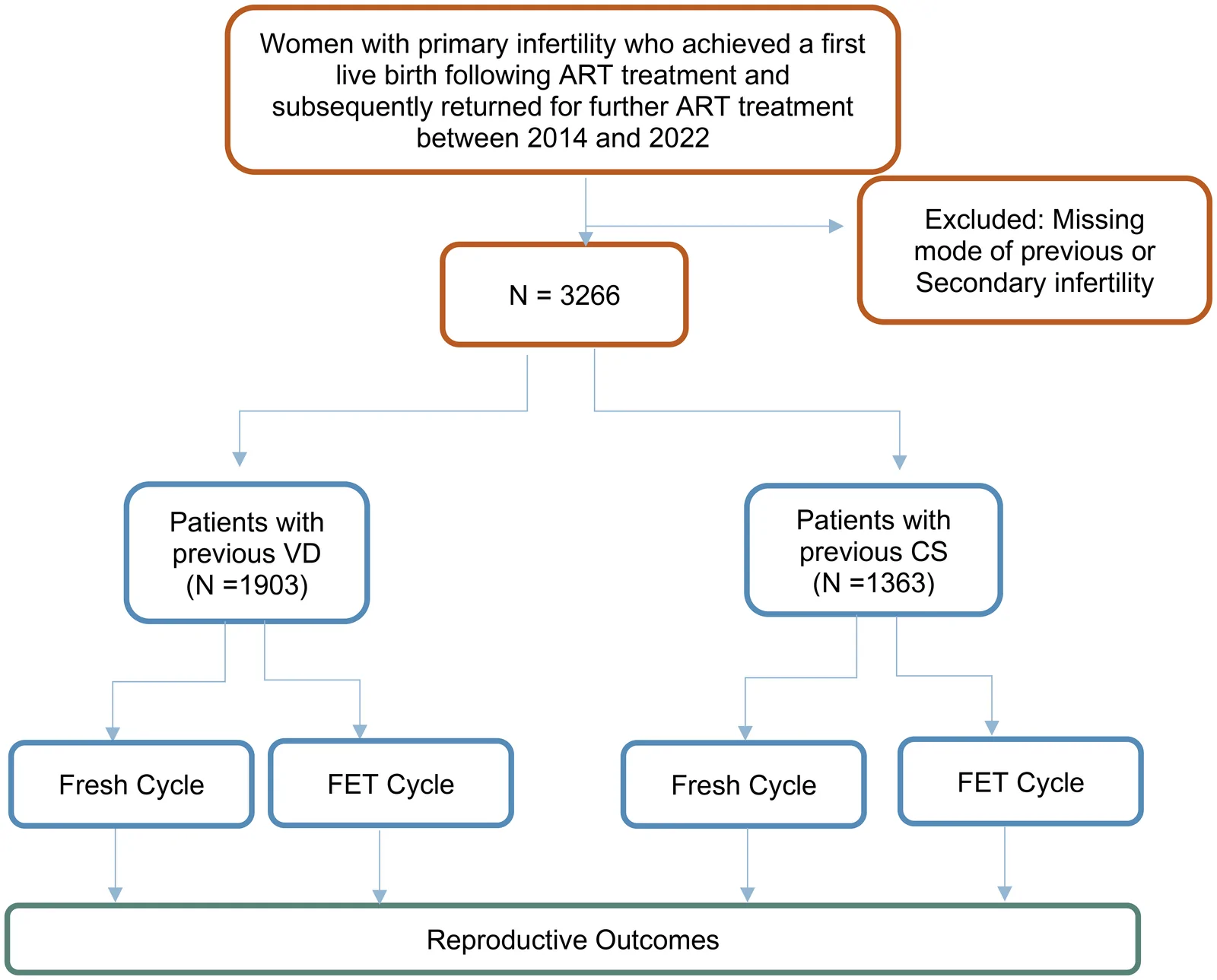
Flowchart of patient selection. This retrospective study included women with primary infertility who achieved a first live birth following ART treatment and subsequently returned for further ART treatment. According to the mode of the previous delivery, women were allocated to the vaginal delivery (VD) or caesarean section (CS) group. Reproductive outcomes following subsequent fresh and frozen embryo transfers were compared between groups. Missing values are reported for variables with incomplete data.

We conducted a retrospective cohort study, collecting a total of 3,266 cases of women who delivered after ART treatment and were registered in the Swiss ART registry (FIVNAT) from 2014 to 2022 (**Figure 1**).

### FIVNAT

The Swiss National IVF Registry, FIVNAT, is a nationwide registry established by the Swiss Society for Reproductive Medicine (SGRM) to systematically monitor assisted reproductive technology (ART) treatments and outcomes in Switzerland. The registry includes all licensed Swiss ART centers and serves as the official national database for quality assurance and surveillance of ART practice. Data are prospectively collected using standardized reporting procedures and include patients’ characteristics, treatment details, pregnancy outcomes, and live birth data. Owing to its comprehensive national coverage and high level of data completeness, FIVNAT-CH has been widely used for epidemiological and retrospective analyses of ART outcomes and trends in reproductive medicine in Switzerland (21–24).

### Study population

We conducted a retrospective cohort study using data from the Swiss national ART registry (FIVNAT) collected between 2014 and 2022.

The study population consisted of women who had initially been diagnosed with primary infertility and underwent ART treatment, resulting in a first live birth. According to the ESHRE definition, primary infertility refers to the inability to achieve a clinical pregnancy after at least 12 months of regular unprotected intercourse in a woman who has never previously achieved a pregnancy (25).

Following this first ART-conceived live birth, women subsequently returned to an ART center for further fertility treatment with the intention of achieving another pregnancy. The interval between the previous delivery and the subsequent ART treatment varied among patients. At study entry, all women therefore had a history of primary infertility, a previous successful ART treatment resulting in a live birth, and at least one subsequent embryo transfer following that delivery.

Women were classified according to the mode of their previous delivery into two groups: (i) previous caesarean section (CSD) and (ii) previous vaginal delivery (VD), including vacuum extraction and forceps-assisted delivery. The objective of the study was to evaluate whether the mode of the previous delivery was associated with the outcome of subsequent ART treatments.

All fresh and frozen–thawed embryo transfers performed after the first live birth were included in the analysis. Outcomes of the first embryo transfer as well as outcomes across all subsequent embryo transfers were analyzed with regard to clinical pregnancy and live birth. Women with secondary infertility or missing information regarding the mode of the previous delivery were excluded.

A total of 3,266 women met the inclusion criteria and were included in the study (1,903 with a previous vaginal delivery and 1,363 with a previous caesarean section). All women were included in the analysis of the first embryo transfer following the previous delivery. For the cumulative analyses, 6,345 embryo transfers were analyzed to evaluate the cumulative probability of clinical pregnancy, and 7,299 embryo transfers were analyzed to evaluate the cumulative probability of live birth. These analyses included all successive embryo transfers performed after the previous delivery until the first clinical pregnancy or live birth, respectively, or until the last available embryo transfer if the outcome was not achieved.

### Outcomes

Primary outcome was the CPR. Clinical pregnancy was defined as an ultrasound-confirmed pregnancy at 6 weeks. Pre-specified secondary outcomes included LBR, miscarriage rate, and maternal and pregnancy outcomes. Live birth was defined as a delivery resulting in a live newborn (25). Miscarriages were defined as induced or spontaneous abortions before 24 weeks of gestation (25). Maternal outcomes included pregnancy complications such as first, second, and third trimester hemorrhage, preterm delivery, preterm rupture of membranes, placenta previa, isolated hypertension (>140/90 mmHg), pre-eclampsia, intrauterine growth retardation, and gestational diabetes. Obstetric outcomes included gestational age and mode of delivery.

### Statistical analysis

The characteristics of the 3,266 included patients were compared according to the mode of delivery (VD vs. CSD). Occurrences were compared using chi-square tests and means using *T*-tests. None of the generated *p*-values was corrected for multiple testing; thus, they are nominal and should be interpreted accordingly. All analyses were performed using SAS 9.4. Multivariable mixed-effects logistic regression models were used to analyze the outcomes of the first embryo transfer cycle. Female age and the number of transferred embryos were included as fixed-effect covariates, while center ID was included as a random effect to account for clustering by ART center. Adjusted odds ratios (aORs) with 95% confidence intervals (CIs) were calculated. For cumulative reproductive outcomes, multivariable mixed-effects Cox proportional hazards models were applied. Female age was included as a fixed-effect covariate and center ID as a random effect. Hazard ratios (aHRs) with 95% confidence intervals (CIs) were estimated. The number of embryo transfers or clinical pregnancies was used as the exposure unit, depending on the outcome analyzed.

#### Analysis of first embryo transfer cycle

The characteristics of the first ET after delivery, as well as maternal and pregnancy outcomes, were evaluated. Multivariate logistic regression analyses were performed to compare outcomes. Odds ratios (OR) were calculated using mixed logit models adjusted for female age and number of embryos transferred until CP or/and LB as fixed effect and center ID as random effect.

#### Analysis of all embryo transfers leading to pregnancy or live birth

Pregnancy or live birth was assessed for each of the 3,266 included patients by all available embryo transfers up to outcome. Hazard ratios (HR) for the outcome of successive ETs were calculated using mixed Cox models adjusted for female age as fixed effect, center ID as random effect, and either number of transfers or number of clinical pregnancies as unit of exposure.

## Results

### Baseline characteristics

A total of 3,266 women with embryo transfers after a previous delivery were included in the study and divided into two groups: VD (*N* = 1,903) and CSD (*N* = 1,363). The mean maternal age was 35.9 and 36.8 years in the VD and CSD groups, respectively. The maternal characteristics in the VD and CSD groups were comparable (**Table 1**). The distribution of ART characteristics was comparable between groups. No significant differences were observed in the type of fertilization (IVF, ICSI, or mixed IVF/ICSI; *p* = 0.318) or in the proportion of fresh versus frozen–thawed embryo transfers (*p* = 0.549), indicating a similar treatment distribution between women with previous caesarean and vaginal delivery (**Table 1**). The distribution of the cumulative number of embryo transfers is presented in **Table 1** and was comparable between women with previous vaginal delivery and previous caesarean delivery.

**Table 1:** Characteristics of the included women and embryo transfers.

Characteristic	Women after vaginal delivery (VD) (*N* = 1,903)	Women after caesarean section delivery (CSD) (*N* = 1,363)	*p*-value
Maternal age, years, mean ± SD			
At follicle aspiration	33.9 ± 3.9	34.8 ± 3.9	**<0.001**
At first embryo transfer	35.9 ± 3.7	36.8 ± 3.7	**<0.001**
Cause of infertility, *n* (%)			
Anovulation/d” “ysovulation/PCOS	312 (16.4)	221 (16.2)	0.89
Endometriosis	217 (11.4)	177 (13.0)	0.171
Tubal factor	197 (10.4)	136 (10.0)	0.728
Increased female age^a^	23 (1.2)	41 (3.0)	**<0.001**
Donor sperm IVF/ICSDI	12 (0.6)	11 (0.8)	0.552
Genetic causes of infertility	4 (0.2)	6 (0.4)	0.241
Male infertility	1,291 (67.8)	894 (65.6)	0.178
Male immunological infertility	9 (0.5)	8 (0.6)	0.655
Other causes of infertility	123 (6.5)	119 (8.7)	**0.015**
Unexplained infertility causes	223 (11.7)	127 (9.3)	**0.029**
Characteristics of first transfer cycle			
Type of oocyte fertilization, *n* (%)			0.318
IVF	205 (10.8)	165 (12.1)	
ICSDI	1,545 (81.2)	1,102 (80.9)	
Mixed	153 (8.0)	96 (7.0)	
Type of transfer, *n* (%)			
Fresh	350 (18.4)	262 (19.2)	0.549
Frozen–thawed	1,553 (81.6)	1,101 (80.8)	
Months from previous delivery to transfer, mean ± SD	20.0 ± 10.1	21.9 ± 10.8	**<0.001**
Number of transferred embryos, *n* (%)			
1	1,561 (82.0)	1,153 (84.6)	0.152
2	331 (17.4)	204 (15.0)	
≥3	11 (0.6)	6 (0.4)	
Number of babies born, *n* (%)			
0	1,282 (67.4)	957 (70.2)	0.368
1	583 (30.6)	381 (28.0)	
2	29 (1.5)	18 (1.3)	
3	1 (0.1)	1 (0.1)	
Unknown	8 (0.4)	6 (0.4)	
Characteristics of all transfer cycles			
From first transfer to clinical pregnancy^a^			
Months from previous delivery to transfer, mean ± SD	23.2 ± 11.4	25.6 ± 12.1	**<0.001**
Cumulative number of transferred embryos, mean ± SD	2.3 ± 2.0	2.4 ± 2.2	0.204
Number of transfers, *n* (%)			0.167
1	1,057 (55.5)	734 (53.9)	
2	430 (22.6)	315 (23.1)	
3	213 (11.2)	142 (10.4)	
4	95 (5.0)	75 (5.5)	
5	41 (2.2)	43 (3.2)	
6	36 (1.9)	16 (1.2)	
7	8 (0.4)	17 (1.2)	
8	8 (0.4)	8 (0.6)	
9	8 (0.4)	5 (0.4)	
≥10	7 (0.4)	8 (0.6)	
From first transfer to live birth^b^			
Months from previous delivery to transfer, mean ± SD	24.7 ± 12.2	26.9 ± 12.5	**<0.001**
Cumulative number of transferred embryos, mean ± SD	2.7 ± 2.3	2.7 ± 2.5	0.482
Number of transfers, *n* (%)			
1	901 (47.3)	640 (47.0)	0.071
2	448 (23.5)	327 (24.0)	
3	254 (13.3)	154 (11.3)	
4	132 (6.9)	95 (7.0)	
5	57 (3.0)	62 (4.5)	
6	51 (2.7)	25 (1.8)	
7	21 (1.1)	24 (1.8)	
8	16 (0.8)	13 (1.0)	
9	10 (0.5)	11 (0.8)	
≥10	13 (0.8)	12 (0.8)	

From the first transfer to the first clinical pregnancy or to the last available transfer if pregnancy is not achieved. Missing values are reported for variables with incomplete data.

^a^ Advanced female age was recorded according to the treating physician’s infertility diagnosis in the Swiss ART registry (FIVNAT); no predefined age threshold was specified in the registry.

^b^ From the first transfer to the first live birth or to the last available transfer if a live birth is not achieved.

Bold values indicate statistically significant differences (p < 0.05).

#### Analysis of first embryo transfer cycle

The results of the first cycle with embryo transfer after delivery (VD vs. CSD) were analyzed and are described in this section. The interval between the previous delivery and the first embryo transfer was significantly shorter in women with a previous VD than in those with a previous CSD (20.0 vs. 21.9 months, *p* < 0.001; **Table 1**). In the unadjusted analysis, CP was significantly more frequent after VD versus CSD (43.4% vs. 38.7%, *p* = 0.008). No significant difference in live birth rate (74.3% vs. 75.8%) or miscarriage rate (24.2% vs. 23.1%) was observed between the two groups (**Table 2**). In the adjusted multivariate logistic regression analysis, differences in CPR (adjusted odds ratio (aOR) = 0.87; 95% CI 0.75–1.01; *p* = 0.067) and LBR (aOR = 0.92; 95% CI 0.78–1.07; *p* = 2.64) were both not significant (**Table 2**).

**Table 2:** Outcomes of the first embryo transfers.

Univariate analysis					
	Women after vaginal delivery (VD) (N = 1903)	Women after caesarean section delivery (CSD) (N = 1363)	p-value		
Outcome of transfer cycles					
Clinical pregnancies, n (%)	825 (43.4)	528 (38.7)	**0.008**		
Live births, n (%)	613 (32.2)	400 (29.3)	0.081		
Outcome of clinical pregnancies	n = 825	n = 528			
Live births, m (%)	613 (74.3)	400 (75.8)	0.451		
Miscarriage, m (%)	200 (24.2)	122 (23.1)	0.665		
Multivariate logistic regression analysis					
	Covariate	Comparator	Reference	Adjusted OR (95 % CI)	p-value
Clinical pregnancies by transfer (n=3261)	Treatment group	CSD	VD	0.87 (0.75-1.01)	0.067
	Age of women, years	40	35	**0.71 (0.65-0.78)**	**<0.001**
	Number of transferred embryos	≥2	1	1.07 (0.88-1.29)	0.51
Live births by transfer (n=3252)	Treatment group	CSD	VD	0.92 (0.78-1.07)	0.264
	Age of women, years	40	35	**0.73 (0.66-0.81)**	**<0.001**
	Number of transferred embryos	≥2	1	0.98 (0.79-1.20)	0.819

OR, odds ratio.

Bold values indicate statistically significant differences (p < 0.05).

#### Analysis of all embryo transfers leading to pregnancy

A total of 6,345 embryo transfers following the previous delivery (VD vs. CSD) were included in the cumulative analysis of clinical pregnancy. The cumulative probability of achieving a clinical pregnancy was significantly higher in the VD group than in the CSD group (73.9% vs. 66.3%; *p* < 0.001). In the multivariable mixed-effects Cox regression analysis, previous caesarean section was associated with a significantly lower cumulative clinical pregnancy rate than previous vaginal delivery (aHR 0.89, 95% CI 0.82–0.97; *p* = 0.009) (**Table 3**).

**Table 3:** Outcomes of all embryo transfers.

Univariate analysis					
	Women after after vaginal delivery (VD) (N = 1903)	Women after caesarean section delivery (CSD) (N = 1363)	p-value		
**Clinical pregnancies, n (%)**	1406 (73.9)	904 (66.3)	**<0.001**		
**Live births, n (%)**	1208 (63.5)	758 (55.6)	**<0.001**		
Multivariate Cox regression analysis					
	Covariate	Comparator	Reference	Adjusted HR (95 % CI)	p-value
**Clinical pregnancies by transfer (n=3257)**	Treatment group	CSD	VD	**0.89 (0.82-0.97)**	**0.009**
	Age of women, years	40	35	**0.80 (0.76-0.85) **	**<.001**
**Live births by transfer (n=3241)**	Treatment group	CSD	VD	**0.89 (0.81-0.97)**	**0.010**
	Age of women, years	40	35	**0.77 (0.73-0.82)**	**<0.001**

HR, hazard ratio.

Bold values indicate statistically significant differences (p < 0.05).

Among the 2,310 clinical pregnancies (VD, *n* = 1,406; CSD, *n* = 904), miscarriage rates were similar in the two groups (24.3% vs. 25.1%). The multivariable Cox regression analysis likewise showed no significant difference in the probability of live birth after clinical pregnancy between the VD and CSD groups (aHR 1.00, 95% CI 0.91–1.10; *p* = 0.982) (**Table 4**).

**Table 4:** Outcomes of all pregnancies.

Univariate analysis					
	Pregnancies (n = 1406) of women (N = 1903) after vaginal delivery	Pregnancies (n = 904) of women (N= 1363) after caesarean section delivery	p-value		
Pregnancy outcomes (n, %)					
Delivery	1048 (74.5)	669 (74.0)	0.259		
Miscarriage < 12 Weeks	313 (22.3)	209 (23.1)			
Miscarriage between 3 and 6 months	16 (1.1)	7 (0.8)			
Ectopic pregnancy	12 (0.9)	2 (0.2)			
Induced abortion	12 (0.9)	11 (1.2)			
Unknown	5 (0.4)	6 (0.7)			
Multivariate cox regression analysis from pregnancy to live birth					
	Covariate	Comparator	Reference	Adjusted HR (95 % CI)	p-value
Live birth by clinical pregnancy (n=3241)	Regimen	CSD	VD	1.00 (0.91-1.10)	0.982
	Age of mother,years	40	35	0.96 (0.90-1.02)	0.2

HR, hazard ratio.

#### Analysis of all embryo transfers leading to live birth

A total of 7,299 embryo transfers following the previous delivery (VD vs. CSD) were included in the cumulative analysis of live birth. The cumulative probability of achieving a live birth was significantly higher in the VD group than in the CSD group (63.5% vs. 55.6%; *p* < 0.001). In the multivariable Cox regression analysis, previous caesarean section was associated with a significantly lower cumulative live birth rate than previous vaginal delivery (aHR 0.89, 95% CI 0.81–0.97; *p* = 0.010) (**Table 3**).

### Pregnancy and obstetric outcomes

In the 1,966 pregnancies leading to live birth (VD = 1,208; CSD = 758), no significant differences were observed in pregnancy pathologies between the groups. In obstetrical outcomes, a significant difference emerged in the delivery mode between the two study groups: the caesarean section rate following ET was 19.8% in the VD and 80.3% in the CSD group (*p* = 0.001) (**Table 5**).

**Table 5:** Pregnancy complications after all embryo transfers and obstetrical outcomes.

Characteristic	Live birth (*n* = 1,208) of women (*N* = 1,903) after vaginal delivery	Live birth (*n* = 758) of women (*N* = 1,363) after caesarean section delivery	*p*-value
Pregnancy pathology (*n*, %)			
Bleeding, 1st trimester	61 (5.0)	43 (5.7)	0.548
Bleeding, 2nd trimester	13 (1.1)	7 (0.9)	0.743
Bleeding, 3rd trimester	11 (0.9)	10 (1.3)	0.391
Cervical insufficiency with cerclage	4 (0.3)	1 (0.1)	0.393
Premature rupture of membrane	30 (2.5)	20 (2.6)	0.832
Premature labour, 2nd trimester	4 (0.3)	4 (0.5)	0.505
Premature labour, 3rd trimester	12 (1.0)	2 (0.3)	0.061
Gestational diabetes	59 (4.9)	39 (5.1)	0.796
Isolated hypertension (>140/90)	6 (0.5)	6 (0.8)	0.414
Preeclampsia	10 (0.8)	7 (0.9)	0.824
Eclampsia	1 (0.1)	1 (0.1)	0.739
Intrauterine growth restriction (IUGR)	11 (0.9)	8 (1.1)	0.749
Placenta previa	14 (1.2)	12 (1.6)	0.423
Cholestasis	2 (0.2)	3 (0.4)	0.324
Hospitalization	32 (2.6)	28 (3.7)	0.19
Other	35 (2.9)	30 (4.0)	0.201
Mode of delivery (*n*, %)			
Spontaneous	909 (75.2)	110 (14.5)	**<0.001**
Forceps	14 (1.2)	7 (0.9)	
Vacuum	40 (3.3)	32 (4.2)	
Caesarean section	239 (19.8)	609 (80.3)	
Unknown	6 (0.5)	0 (0)	

Bold value indicate statistically significant differences (p < 0.05).

## Discussion

Our study revealed three clinically relevant findings. First, women with a previous caesarean section had lower cumulative clinical pregnancy and live birth rates following ART. Importantly, this association was primarily observed in cumulative analyses across multiple embryo transfers, whereas differences after the first embryo transfer were no longer statistically significant after adjustment for confounding variables. Second, miscarriage rates were similar between groups, suggesting that any potential effect of previous caesarean section may occur before or during implantation rather than during the later stages of pregnancy. Third, no significant differences in pregnancy-related complications were observed, although these findings should be interpreted cautiously because of the possibility of underreporting in registry-based data.

The World Health Organization (WHO) has estimated the optimal rate of CSD to be 15% (26). However, the proportion of CSD in Switzerland is 33.0% (4). The increasing prevalence of CSD highlights the necessity for further investigation into the long-term complications associated with CSD scarring (5, 27, 28), particularly in regard to its impact on subsequent fertility.

This nationwide registry study provides contemporary evidence on the reproductive consequences of previous caesarean delivery in women undergoing ART. Our first finding, the negative effect of previous CSD on reproductive outcomes following ART, is in line with most previous studies but has hereby been confirmed with a large and more recent set of data. The women in the CSD group have a significantly higher maternal age of around 1 year at the time of OPU (VD: 33.9 ± 3.9 vs. CSD: 34.8 ± 3.9; *p* ≤ 0.001) and at the time of ET (VD: 35.9 ± 3.7 vs. CSD: 36.8 ± 3.7; *p* ≤ 0.001). This could potentially impact oocyte quality and create a bias in the unadjusted data. However, as the number of previously used embryos was the same in both groups, this bias can be assumed to be very limited.

Wang et al. (29) analyzed in a retrospective cohort study 310 patients undergoing ART cycles with fresh embryo transfers. They found lower implantation rates (24.0% vs. 34.6%, *p* ≤ 0.05) and clinical pregnancy rates (40.2% vs. 54.2%, *p* ≤ 0.05) in patients with previous CSD (29). Huang et al. (30) studied the effect of previous CSD on the pregnancy outcomes of ART with frozen–thawed embryo transfer cycles (FET). Their findings indicate that women with a history of CSD have significantly lower CPR and LBR compared to those with previous VD, with CPR of 38.3% vs. 44.5% (*p* = .005) and LBR of 27.5% vs. 33.4% (*p* = 0.003). Adjusted analyses maintained this association, showing an adjusted odds ratio (OR) of 0.80 (95% CI 0.66–0.96) for CPR and 0.78 (95% CI 0.63–0.95) for LBR. Women with previous CSD had an adjusted OR 1.47 (95% CI 1.01–2.14) for an early miscarriage.

A meta-analysis, published several years ago in 2013, included 18 studies with 591,850 patients and demonstrated that CSD reduced the likelihood of subsequent pregnancy by an average of 9% compared with VD, suggesting a potential long-term impact of delivery mode on fertility outcomes (5).

However, not all studies found a reduced fertility following CSD. Zhang et al. (2022) performed a small retrospective study with a total of 231 patients and could not detect a negative impact of CSD on embryo implantation rate and pregnancy outcome after FET (31). The findings of Zhang et al. may be an exception due to methodological or population differences, requiring larger studies for confirmation. Our observed association may also have been influenced by unmeasured confounders, including patients’ characteristics, embryo-related factors, and treatment-specific variables that were not available in the registry and could therefore not be incorporated into the adjusted analyses.

Another recent meta-analysis by Vitagliano et al. (32) published in 2024 included eight studies with 10,873 patients and found that isthmocele, rather than CSD *per se*, is the factor that affects fertility. Pregnancy rates in women with a previous CSD were similar to those with VD, whereas pregnancy rates were lower in women with an isthmocele than in those without an isthmocele. A subgroup analysis showed that intrauterine fluid, also called serometra, negatively affects pregnancy rates in women with isthmocele.

This finding raises the question on whether the impact of CSD on fertility is due to the uterine incision and scar and might therefore affect any women or if it is due to specific individual pathologies on top of the uterine scar, such as the formation of an isthmocele or the accumulation of intrauterine fluid which occurs only in a specific subset of women. The uterine incision might have healed insufficiently, resulting in alterations of the uterine anatomy, which may lead to the formation of a uterine scar defect of varying magnitude, described as isthmocele (6). The routine use of ultrasound scanners with high-resolution vaginal ultrasound probes has facilitated the straightforward detection of isthmoceles (32, 33).

Potential hypotheses about the effect of isthmocele on fertility outcomes have been summed up by Vissers et al. (12). First, the accumulation of fluid or blood in the uterine cavity could affect embryo viability and implantation through exposure to embryotoxic factors. Second, altered immunobiology and/or increased inflammatory activity around the uterine scar and isthmocele may decrease implantation rate. The third hypothesis is based on uncoordinated or altered uterine contractions following CSD. The uterine scar forms a functional barrier of muscular activity in the lower segment of the uterus and therefore affects uterine function. This may result in impaired uterine contractility around the scar. Fourth, the embryo transfer catheter might be more difficult to insert due to a distorted cervical canal, especially in case of an isthmocele in combination with a retroflexed uterus (6).

Some studies have tried to address these suggested causes of lower pregnancy rates caused by previous CSD. Vissers et al. (2023) also analyzed the impact of CSD on the outcomes of 1,317 ART cycles. They found that the LBR was significantly lower in women with a prior CSD compared to those with a previous VD, with rates of 15.9% and 23.3%, respectively (OR 0.63, 95% CI 0.45–0.87) (14). Lower rates of ongoing pregnancy, clinical pregnancy, and biochemical pregnancy were also observed. Notably, the LBR in CSD group was even lower at 10.7%.

Wang et al. (2020) revealed that in cases with CSD in which a serometra was ultrasonographically detected, the pregnancy rate was as low as 12.5% (29). However, these results were not adjusted for confounding factors, which is considered a limitation of the study. Zhao et al. (2021) also analysed if CSD can lead to technical problems in case of embryo transfer in ART. They described much higher technical difficulties of embryo transfer in women having had a CSD compared to those with a VD (34) (RR = 8.23; 95% CI 4.63–14.65; *p* ≤ 0.001).

Recent evidence suggests that reproductive outcomes after caesarean section may also be influenced by the surgical procedure itself and the quality of uterine healing. The location of the uterine incision, the technique used for uterine closure, and the subsequent remodeling of the myometrium may affect long-term uterine function and reproductive performance. Experimental and clinical data indicate that optimal restoration of uterine anatomy during hysterotomy repair is associated with improved scar integrity and may reduce the risk of long-term complications, including scar defects and impaired fertility. Therefore, the observed association between previous caesarean section and lower cumulative pregnancy rates may reflect not only the presence of isthmocele but also broader effects of surgical technique and scar healing on uterine function. Furthermore, the longer interval between delivery and subsequent ART treatment observed after caesarean section may partly reflect clinical recommendations advising women to postpone conception for approximately 12 months following surgery to allow adequate uterine healing. Therefore, the prolonged time to subsequent embryo transfer observed in the CSD group may not solely reflect reduced fertility but may also be influenced by clinical practice patterns following caesarean delivery. Consequently, future prospective studies should evaluate not only the presence of isthmocele but also the surgical characteristics of the primary caesarean section, including uterine closure techniques and scar healing parameters, to better understand the mechanisms underlying the observed association.

The second clinically relevant finding of our study is that a CSD did not affect the clinical miscarriage rate. This finding is in contrast with a systematic review and meta-analysis published in 2021, including 4,084 studies with 1,524,695 women, which showed a higher rate of miscarriage (RR = 1.39; 95% CI 1.18–1.64; *p* ≤ 0.0001) (35). The contrasting results of both studies could be attributed to different time points of ultrasound analysis. As we defined miscarriage rate only as miscarriages after ultrasound detection of an amniotic sac or an embryo, other studies might have found different results due to very early detection of miscarriages. The absence of differences in miscarriage rates should be interpreted cautiously because registry-based datasets may incompletely capture biochemical pregnancies and very early pregnancy losses. In addition, some pregnancy outcomes may have been underreported or classified as unknown.

The third relevant finding of our study was that we did not find any statistically significant differences regarding pregnancy-related pathology. This is also in contrast with a meta-analysis published in *The Lancet* in 2020, including 27 studies with 1,524,695 women. It was shown that pregnancies following a CSD were associated with an increased risks of adverse maternal outcomes, such as uterine rupture, placenta previa, and hemorrhage. Therefore, the authors recommended that future deliveries after CSD need to be monitored, given the potential risks to maternal and infant health (35).

The reasons for the differences of both studies might be manifold. They might be due to different CSD technologies as the meta-analysis included CSDs performed as early as 1975. Furthermore, different definitions of obstetrical complications and limitations in data documentation in the Swiss ART registry were noted. The ART registry reports lower pregnancy complication rates than expected from published prevalence, suggesting possible non-reporting of obstetric complications.

### Strengths and limitations

The major strength of our study is the large cohort (*n* = 3,266) of ART-treated patients representing all Swiss ART data from 2014 to 2022.

Furthermore, the registry contains standardized information on ART treatments, pregnancy outcomes, and live births collected over a prolonged observation period, allowing the assessment of long-term reproductive outcomes following a previous delivery. Finally, the availability of cumulative ART data enabled us to distinguish between outcomes after the first embryo transfer and cumulative outcomes across successive transfers, providing a more comprehensive evaluation of reproductive performance after caesarean section and vaginal delivery.

However, several limitations should be considered when interpreting our findings. First, this was a retrospective registry-based study, and therefore causal relationships cannot be established. Second, some potentially important confounding factors were not available in the Swiss ART registry. In particular, data on body mass index (BMI), indication for caesarean section, uterine closure techniques, cleavage-stage transfer, transfer difficulty, endometrial preparation, scar defect characteristics, and intrauterine fluid were not recorded and could therefore not be included in the analyses (36, 37). This is particularly relevant because factors such as BMI and surgical characteristics may influence uterine healing and the subsequent development of scar-related abnormalities. Another weakness is the missing data on the indication of the CSDs and the suture techniques used. Therefore, we could not assess the role of related factors contributing to the risk of developing an isthmocele. Furthermore, obstetrical outcomes may be subject to missing data and non-reporting. The lower prevalence of pregnancy complications recorded in the registry compared with published estimates suggests incomplete ascertainment of obstetric outcomes; therefore, findings regarding miscarriage and pregnancy-related complications should be interpreted with caution. Furthermore, the Swiss ART registry does not capture complete reproductive histories outside registered ART treatments. Therefore, information on previous spontaneous pregnancies and the total number of previous deliveries, including repeated caesarean deliveries, was unavailable.

## Conclusion

Our study confirmed that CSD reduces CPR and consequently LBR following ART. In contrast to other studies, clinical miscarriage rate was not increased, indicating that the main impact of CSD on CPR and LBR occurs at a very early stage of pregnancy, around the time of implantation. Furthermore, we could also not find more prevalent pregnancy complications. Even though our study substantially contributes to this clinically relevant and partly controversially described topic, it also demonstrates that further prospective and well-controlled studies focusing on potential causes for the detrimental effect of CSD, such as the size of isthmoceles, intrauterine fluid formation, and even the indication and surgical technique of the CSD, are urgently required to possibly decrease the risk of infertility.

## Data Availability

The original contributions presented in the study are included in the article/supplementary material. Further inquiries can be directed to the corresponding author.
